# Jejunal intussusceptions due to metastatic malignant melanoma: a case report

**DOI:** 10.1093/jscr/rjaa553

**Published:** 2021-01-18

**Authors:** Tiago Correia de Sá, Ana Isabel Ferreira, Filomena Barreto, Mariana Santos

**Affiliations:** General Surgery Department, Centro Hospitalar Tâmega e Sousa, 4564-007, Guilhufe, Portugal; General Surgery Department, Centro Hospitalar Tâmega e Sousa, 4564-007, Guilhufe, Portugal; Anatomic Pathology Department, LAP/UNILABS, 4250-170, Porto, Portugal; General Surgery Department, Centro Hospitalar Tâmega e Sousa, 4564-007, Guilhufe, Portugal

## Abstract

Intussusception is an infrequent cause of mechanical bowel obstruction in adults and surgical resection is warranted in most cases. Small bowel is a common site of recurrence from cutaneous melanoma but early diagnosis is still a challenge. Acute peritonitis, haemorrhage and obstruction are known clinical presentations. Wide surgical excision with free margins and accompanied mesentery is the treatment of choice and may improve the prognosis. We present a case of small bowel obstruction due to three intussusceptions by metastatic malignant melanoma submitted to surgery.

## INTRODUCTION

Bowel intussusception, an infrequent cause of mechanical intestinal obstruction in adults, is the telescoping of a proximal segment of the gastrointestinal tract into an adjacent distal segment [1]. Up to 90% of intussusceptions in adults have an identifiable aetiology and the most common affected site is the small bowel. Approximately a third of the cases are malignant and include carcinoid tumours, leiomyosarcomas, adenocarcinomas, lymphomas, metastatic lesions, among others [1].

Intestinal melanomas can be primary tumours, although they are extremely rare and by many considered metastatic from an unknown cutaneous melanoma, or metastasis of cutaneous, ocular or anal melanomas. Around 60% of the patients who die from melanoma have gastrointestinal metastasis, but only ~5% are detected before death [2]. Metastatic lesions in the small bowel are frequently multiple, and the presence of ulceration is common. The time between primary tumour excision and small bowel metastasis ranges from 6 months to 90 months [2]. Small bowel intussusception caused by metastatic melanoma is a very rare condition.

We present a case of small bowel obstruction by three intussusceptions caused by jejunal metastasis of malignant melanoma.

## CASE REPORT

We report a case of a cutaneous malignant melanoma which metastasised to the small bowel causing three jejuno-jejunal intussusceptions.

**Figure 1:**
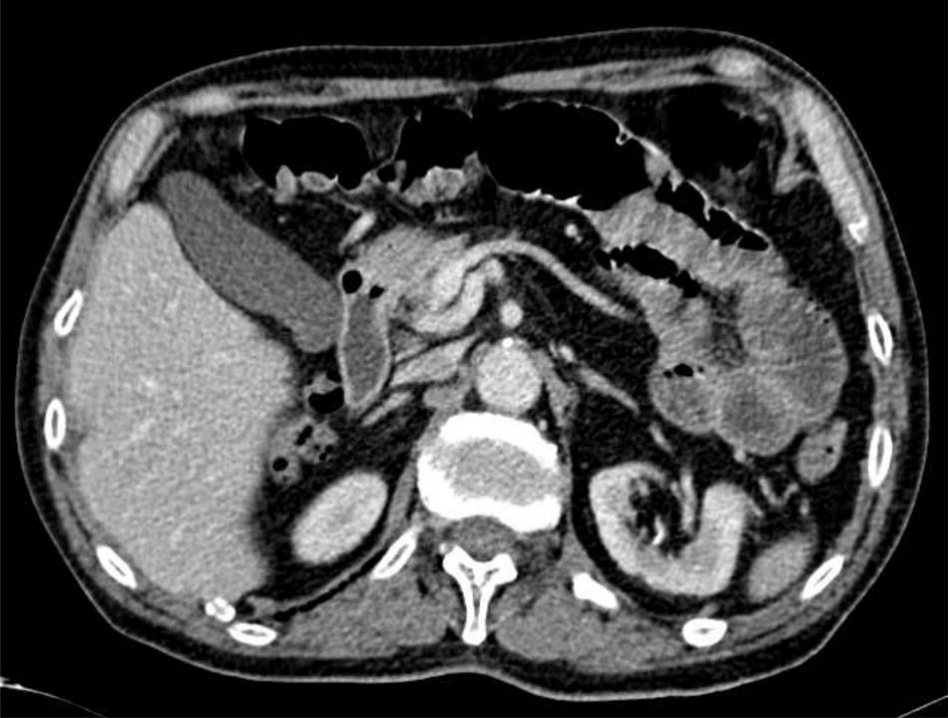
CT scan targetlike finding. Contrast enhanced CT scan of the abdomen demonstrates the typical multi-layered appearance of a small bowel intussusception. The intussusceptum is accompanied by a mesenteric fat and blood vessels and surrounded by the thick-walled intussuscipiens, the target-like finding, pathognomonic for intussusception.

A 82-year-old male presented to our Emergency Department with a history of colicky abdominal pain in the upper quadrants, exacerbated by food and vomiting, suggestive of bowel obstruction. The patient referred intermittent attacks of non-specific abdominal pain over the last weeks. Routine laboratory tests revealed a mild leucocytosis and C-reactive protein elevation, with no further changes. Plain abdominal X-rays revealed multiple dilated loops of small bowel with gas-fluid levels and a subsequent computed tomography (CT) scan confirmed the previous findings, with an area suggestive of small bowel intussusception in the proximal jejunum (Fig. 1).

On direct questioning the patient presented a history of excision of a malignant cutaneous melanoma in the right pre-auricular area 2 years ago, and a superficial parotidectomy due to recurrence 10 months later. The patient maintained regular follow-up, with no evidence of local re-recurrence or metastasis. The patient refused adjuvant therapy, as proposed in the melanoma multidisciplinary meeting.

**Figure 2:**
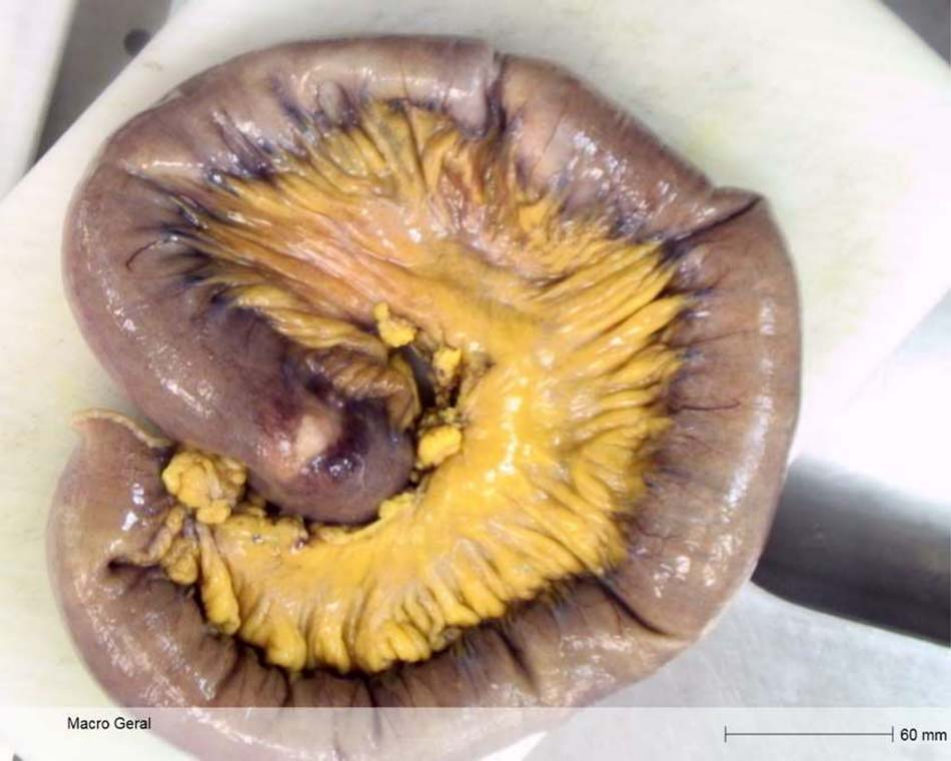
Macroscopic specimen examination. Final anatomopathological examination revealed 60 cm long enterectomy specimen, with 4 polypoid lesions, which caused in areas retraction of the serosa (at 10.5 cm, 23.5 cm and 54 cm from the proximal margin).

The patient was operated after appropriate preparation and, intraoperatively, three intussusceptions of the jejunum at approximately 40, 70 and 100 cm from the Treitz angle were identified (Fig. 2). Full laparotomy did not reveal any other suspicious lesions. The affected small bowel and accompanied mesentery were resected, followed by a latero-lateral isoperistaltic mechanical jejuno-jejunal anastomosis.

**Figure 3:**
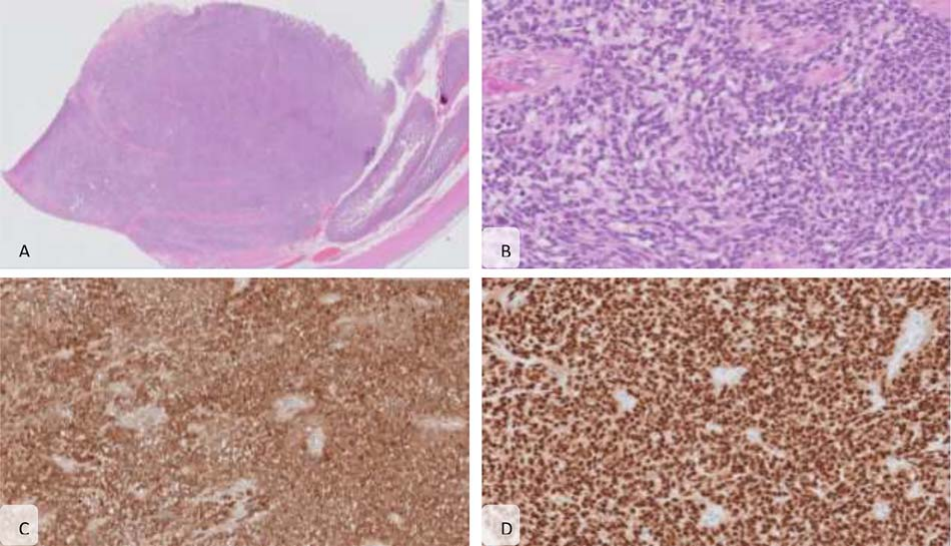
Microscopic features. (**A**) The lesion shows mural and diffuse infiltration of atypical cells (haematoxylin and eosin (H&E), x20) (**B**) forming nests, arranged around vascular structures with papillary-like growth; the cells present big ovoid nuclei with vesicular chromatin and occasional nucleoli and no pigment was seen (H&E, x200); immunohistochemical staining showed that the malignant cells were diffuse positive for PS100 (**C**) and SOX10 (**D**), characteristic of melanoma.

Pathology report identified four polypoid and ulcerated lesions: the first at 3 cm of the proximal margin of the specimen, the second at 10.5 cm from the proximal margin, not identified intra-operatively, the third at 23.5 cm from the proximal margin and a forth lesion 6 cm from the distal margin. On histology examination, the lesions had all the same pattern and an intramural growth, in some point with serosa break. It could be seen an expansive solid growth, with a vague pattern in nests, sometimes pseudopapillary, arranged around vascular structures. The cells were medium-to-large-sized, had a large ovoid nuclei with vesicular chromatin and occasional nucleoli. The cytoplasm was eosinophilic or occasionally clear. There was no pigment. There was numerous mitosis and apoptosis, as necrosis. Lymphovascular invasion was present (Fig. 3). The resection margins and the retrieved 21 lymph nodes were free from tumor invasion. Immunohistochemistry revealed the presence of tumor cells that were positive for the melanoma markers such as SOX10, HMB45 and S100 protein. Based on the patient’s previous medical history, presence of multiple lesions and morphologic aspects together with immunohistochemistry the diagnosis of metastatic melanoma was established.

The patient had an uneventful recovery and was discharged from the hospital on postoperative day five. Regular follow-up was maintained in the first 3 months after surgery. The patient died after by community acquired pneumonia.

## DISCUSSION

Up to 90% of intussusceptions in adults have an identifiable aetiology, and while 60% can be benign, most warrant surgical excision [1].

Small bowel is a common site of gastrointestinal metastatic cutaneous melanoma. Symptom-free period between surgical excision of the primary tumour and small-bowel metastasis is between 6 and 90 months [2]. In this case, the symptom-free period lasted 24 months. Acute peritonitis, haemorrhage and obstruction are described clinical presentations, and the ileum is the most common affected area [2]. However, bowel intussusception is a rare presentation form [2–4]. To our knowledge, this is the first reported case of three jejuno-jejunal intussusceptions due to metastatic malignant melanoma.

Wide surgical resection of the lesions, with sufficient free margins, and accompanied mesentery to remove the lymph nodes is the treatment of choice [2, 3, 5]. Morbidity and mortality are very low [2]. Although this is not a curative procedure and the prognosis is poor, extended surgical resection is the only option and might improve the survival. Currently, 5-year survival rates of up to 40% and disease free survival of up to 10 years have been reported. [2, 6, 5]. No standard systemic therapies are available for multiple melanoma metastasis, including gastrointestinal metastasis, and these cases should be included in clinical trials and subject of multidisciplinary discussion and decision [7]. Completion of the remainder of the adjuvant therapy or postoperative chemotherapy and/or immunotherapy is sometimes administered [2, 8]. Furthermore, in the elderly, melanoma management exhibits special features and a shared-decision making should be pursued, making sure the patient understands the benefits and harms of the proposed treatment. This patient refused all the proposed adjuvant treatments since the first surgical intervention [9].

Despite advances in diagnostic techniques, detection of small-bowel melanoma metastasis is a challenge and most present late in the progression of the disease, resulting in a poor prognosis. A high level of suspicion for metastatic disease is needed in these patients.

## CONFLICT OF INTEREST STATEMENT

None declared.

## FUNDING

None.
